# Exercise: A Possibly Effective Way to Improve Vitamin D Nutritional Status

**DOI:** 10.3390/nu14132652

**Published:** 2022-06-27

**Authors:** Jinghua Zhang, Zhen-Bo Cao

**Affiliations:** 1 Shanghai Frontiers Science Research Base of Exercise and Metabolic Health, Shanghai University of Sport, Shanghai 200438, China; zhangjinghua429@163.com; 2 School of Kinesiology, Shanghai University of Sport, Shanghai 200438, China

**Keywords:** vitamin D, endurance exercise, resistance exercise, adipose tissue, skeletal muscle

## Abstract

Vitamin D deficiency has become a widespread public health problem owing to its potential adverse health effects. Generally, the nutritional status of vitamin D depends on sunlight exposure and dietary or supplementary intake. However, recent studies have found that exercise can influence circulating 25(OH)D levels; although, the results have been inconclusive. In this review, we focused on the effect of exercise on circulating vitamin D metabolites and their possible mechanisms. We found that endurance exercise can significantly increase serum 25(OH)D levels in vitamin D-deficient people but has no significant effect on vitamin D-sufficient people. This benefit has not been observed with resistance training. Only chronic endurance exercise training can significantly increase serum 1,25(OH)_2_D, and the effect may be sex-dependent. Exercise may influence 25(OH)D levels in the circulation by regulating either the vitamin D metabolites stored in tissues or the utilization by target tissues. The effects of exercise on 25(OH)D levels in the circulation may be dependent on many factors, such as the vitamin D nutritional status, exercise type and intensity, and sex. Therefore, further research on the effects and mechanisms of exercise on vitamin D metabolites is required.

## 1. Introduction

The recent increase in vitamin D-related research has led to the discovery of the vitamin D receptor (VDR) in many tissues. A growing body of literature has shown that the biological role of vitamin D goes beyond the traditionally understood duties dealing with muscles and bones and is important for energy metabolism, oxidative stress, maintenance, and improvement of physical fitness [1,2,3,4]. A study found that vitamin D_3_ supplementation increased serum 25(OH)D levels; additionally, the expression of 291 genes, involving as many as 160 metabolic pathways, was significantly upregulated or downregulated [5]. This finding suggests that vitamin D plays an important role in health. Alarmingly, a survey found that vitamin D deficiency has become a global public health problem [6,7]. Vitamin D deficiency is closely associated with various chronic non-communicable diseases and functional disorders [1,8,9,10]. Therefore, maintaining adequate vitamin D levels is significant for promoting health.

Vitamin D is mainly synthesized by the skin, and its sources in food are scarce [11,12]. In the epidermis, 7-dehydrocholesterol can be transformed into vitamin D_3_ upon exposure to sunlight, while vitamin D_2/3_ in foods/supplements is absorbed into the circulation through the intestines. Both skin-synthesized vitamin D_3_ and food/supplement-derived D_2/3_ are catalyzed by 25-hydroxylase [mainly cytochrome P450 family 27 subfamily A member 1 (CYP27A1) and cytochrome P450 family 2 subfamily R member 1 (CYP2R1)] to 25(OH)D in the liver. Due to its long half-life [13] and strong vitamin D binding protein (VDBP) binding ability [14], serum 25(OH)D is the most abundant and stable vitamin D metabolite in the circulation; hence, its serum concentration is used to evaluate the nutritional status of vitamin D [15]. Subsequently, 25(OH)D is catalyzed by 25(OH)D-1α hydroxylase, cytochrome P450 family 27 subfamily B member 1 (CYP27B1), to 1,25(OH)**_2_**D in the kidney, which can bind to the VDR in the target tissue and regulate physiological processes. Vitamin D 24-hydroxylase, cytochrome P450 family 24 subfamily A member 1 (CYP24A1), an important degrading enzyme, can degrade 25(OH)D and 1,25(OH)_2_D in the kidney, which is then excreted through bile. Therefore, it is generally believed that skin synthesis and dietary intake/supplement are the main factors determining the nutritional status of vitamin D. Although scholars have suggested that vitamin D nutritional status can be improved by increasing the duration of sun exposure, the proportion of participants in one study with sufficient sunlight exposure was only 56.12% [16]. Moreover, prolonged UV exposure leads to hyperpigmentation and possible skin cancer [17]. Additionally, vitamin D is a photolabile; thus, prolonged UV exposure does not significantly increase vitamin D levels [18,19]. Therefore, support for prolonged sun exposure has lessened [20]. Vitamin D supplementation is a generally accepted method for improving its nutritional status; however, the recommended standards vary widely among the WHO, IOM, UK, EU, and China [21,22,23]. Moreover, while low doses are considered ineffective, high doses may lead to adverse effects, such as severe hypercalcemia and hyperphosphatemia [24].

Recent studies have found that physical inactivity is an important risk factor for morbidity and mortality from chronic non-communicable diseases [25] and for vitamin D deficiency [26]. Many observational studies have shown that the maintenance of vitamin D nutritional status is related to physical activity/exercise habits [27,28], and physical activity levels are significantly positively correlated with 25(OH)D levels [29,30]. Our meta-analysis of these studies revealed that physical activity was significantly positively correlated with circulating 25(OH)D levels [31]. However, results from experimental studies are inconsistent and may depend on the exercise type [31]. In this review, we focus on endurance exercise and resistance exercise to examine the effect of exercise on 25(OH)D and 1,25(OH)_2_D and its possible mechanisms.

## 2. Endurance Exercise and Vitamin D

### 2.1. The Effect of Acute Endurance Exercise

#### 2.1.1. Human Studies

Five human studies have investigated the effects of acute endurance exercise on 25(OH)D and 1,25(OH)_2_D, with three focusing on professional athletes and two on non-athletes (Table 1). Mieszkowski et al. found that serum 25(OH)D levels in male runners [baseline serum 25(OH)D level >20 ng/mL] with and without vitamin D supplementation were significantly increased immediately and 24 h after an ultra-marathon race compared with those before the race [32]. Dzik also reported that serum 25(OH)D_3_ levels in male soccer players (10–14 years old) significantly increased at 15 min and 1 h after a VO_2_max test [25(OH)D >70 nmol/L]. In an analysis of both pre-pubertal and pubertal boys, the concentration of 25(OH)D_3_ increased 15 min after the VO_2_max test and dropped one hour after exercise, but these changes were not significantly different at specific time points [33]. However, Maimoun found that intensity exercise [47% Wmax, baseline serum 25(OH)D level: 79.4 ± 13.7 nmol/L; 64% Wmax, baseline serum 25(OH)D level: 83.4 ± 16 nmol/L] did not alter the concentration of 25(OH)D in male competitive road cyclists during cycling exercise or after 15 min of recovery [34]. Conversely, two studies involving non-athletes demonstrated that acute endurance exercise may increase circulating 25(OH)D levels. Maimoun et al. found that maximal incremental exercise can significantly increase the level of 25(OH)D in physically highly active elderly participants but not in moderately active elderly and young physically active adults [35]. Sun et al. found that serum 25(OH)D concentration significantly increased immediately and 1, 3, and 24 h after 30 min of cycling exercise at 70% VO_2_peak [36]. However, in the subgroup analysis, the 25(OH)D level increase of women [baseline serum 25(OH)D level:55.1 ± 15.6 nmol/L] was significant only at 24 h after exercise. The acute effect of exercise on 25(OH)D levels may be affected by intensity [33], sex [36], and age [33,35]. As for serum 1,25(OH)_2_D levels, no significant variation was observed in response to acute endurance exercise [34,35,36].

#### 2.1.2. Animal Studies

Results from animal studies investigating the effect of acute endurance exercise on serum 25(OH)D have been inconsistent compared to those from human studies (Table 1). Makanae et al. found that acute endurance exercise (anaerobic threshold intensity) did not alter serum 25(OH)D levels in adult male Sprague-Dawley rats [37]. Moreover, serum 25(OH)D levels in horses were significantly reduced at 30 min, 1 week, and 3 weeks after high-intensity exercise [38]. However, only two experimental animal studies have been conducted to investigate the effect of acute endurance exercise on serum 25(OH)D levels.

### 2.2. The Effect of Chronic Endurance Exercise Training

#### 2.2.1. Human Studies

Twelve human studies investigated the effect of endurance exercise training on serum 25(OH)D levels and did not yield consistent results (Table 1). Some studies found that chronic endurance exercise training can significantly increase serum 25(OH)D levels [39,40,41,42,43,44,45], but other studies have reported contradicting results [46,47,48,49,50]. However, when we sorted these studies, we found that in people with vitamin D deficiency {25(OH)D < 20 ng/mL or 50 nmol/L [57]}, endurance training can significantly improve serum 25(OH)D levels [39,40,42,43,45], and even severe vitamin D deficiency status (<10 ng/mL) improved to vitamin D deficiency status (10–20 ng/mL) in postmenopausal women [42]. However, endurance exercises had no significant effects on serum 25(OH)D levels in overweight and obese subjects, regardless of vitamin D nutritional status [48]. For participants with sufficient vitamin D levels {25(OH)D ≥ 20 ng/mL or 50 nmol/L [57]}, endurance training combined with vitamin D supplementation significantly increased serum 25(OH)D levels [40,44], while endurance training alone did not [40,46,49,50]. While Pilch et al. found that serum 25(OH)D levels were significantly reduced in postmenopausal obese women with sufficient vitamin D levels after endurance exercise intervention, the study was conducted in late autumn and had no control group; hence, it was impossible to determine whether the decrease in 25(OH)D was due to endurance exercise training or a seasonal decline [47]. When considering sun exposure, we found that studies providing relevant sun exposure information were all conducted in the morning [39,40,41,47] or evening [39] or autumn and winter [46,50]. During this time, sun exposure is weaker and has less effect on vitamin D. Taken together, the effect of chronic endurance exercise training on 25(OH)D levels in the circulation may be affected by the vitamin D status.

#### 2.2.2. Animal Studies

Six animal studies investigated the effect of chronic endurance exercise training on 25(OH)D and 1,25(OH)_2_D (Table 1). Aly et al. found that a 4-week swimming regimen significantly increased serum 25(OH)D levels in diabetic mice. No significant change in serum 25(OH)D levels was observed in healthy mice; however, their serum 25(OH)D levels were significantly higher [51]. Buskermolen et al. found that although 6 weeks of endurance training increased serum 25(OH)D levels in female Wistar rats, the change was not significant [52]. The female Wistar rats in Buskermolen’s study were fed 1.5 IU/g (>1000 IU/kg [58,59,60]) vitamin D_3_, which is sufficient to maintain an adequate vitamin D status. Some animal studies have shown that chronic endurance exercise training can significantly increase serum 1,25(OH)_2_D levels in healthy female rats [53,54]. However, while Wang et al. found that 12 weeks of treadmill endurance exercise training slightly increased serum 1,25(OH)_2_D_3_ levels in aged male rats, the results were not significant [55]. Conversely, Xu et al. found that 8 weeks of swimming and downhill running significantly reduced serum 1,25(OH)_2_D_3_ level in 5-week old male mice [56]. In all animal studies which reported increased 1,25(OH)_2_D levels [53,54], the mice were all female, while those that were unchanged or decreased were male [55,56]. The effect of exercise training on 1,25(OH)_2_D may therefore depend on sex.

### 2.3. Mechanism

Endurance exercise induces greater improvements in aerobic capacity and its associated cardiopulmonary and metabolic variables [61]. In terms of energy metabolism, endurance exercise can activate several secondary signal molecules, such as AMPK, CaMKII, and p38, which promote an increase in PGC-1α. Subsequently, PGC-1α promotes mitochondrial biogenesis, exercise-induced fast-to-slow fiber-type transformation, and exercise-induced expression of important muscle antioxidant enzymes. Therefore, endurance exercise, especially submaximal endurance exercise, effectively increases fat metabolism [62]. Adipose tissue is one main storage depot for vitamin D [63]. Hengist et al. suggested that release of vitamin D stored in adipose tissue is a byproduct of lipolysis [64]. In other words, in the process of releasing triglycerides from adipocytes through the action of lipolytic enzymes, the stored vitamin D metabolites were also released. Lipolysis is regulated by various factors, such as atrial natriuretic peptides (ANPs), brain natriuretic peptides (BNPs), insulin, and beta adrenergic hormones [65]. Endurance exercise can promote the release of these hormones [66], promoting lipolytic processes and releasing vitamin D metabolites from the adipose tissue. Moreover, a systematic review showed that all exercise protocols (high-intensity interval exercise, moderate-intensity continuous exercise, and sprint interval exercise) can generate elevated energy expenditure through excessive post-exercise oxygen consumption (EPOC) [67]. Exercise-induced energy deficit has the most potent effect on endogenous lipid metabolism, elevating plasma triacylglycerol concentration and increasing plasma fatty acid mobilization and oxidation the day after performing endurance exercises [68]. The reason that endurance training can increase serum 25(OH)D levels may be attributed to lipolytic processes during exercise and EPOC.

Abboud et al. found that serum 25(OH)D levels in pasture sheep at the end of winter were significantly lower than those during the summer, but intramuscular 25(OH)D content at the end of winter was significantly higher [69]. After 25(OH)D_3_ supplementation, intramuscular 25(OH)D_3_ levels decreased as serum 25(OH)D_3_ levels rose, returning serum and skeletal muscle 25(OH)D concentrations to the more adequate summer levels [25(OH)D > 50 nmol/L] [69]. Abboud et al. found that when the vitamin D nutritional status improves, skeletal muscle cells may lose their ability to accumulate large amounts of 25(OH)D [69]. In addition, vitamin D nutritional status is regulated by a variety of factors such as serum Ca^2+^, Pi, parathyroid hormone (PTH), and FGF23 (fibroblast growth factor-23 (FGF23)) [70,71]. PTH stimulates the expression of CYP27B1 in the kidney, while FGF23, high Ca^2+^ or Pi levels, and 1,25(OH)_2_D downregulate it. In contrast, 1,25(OH)_2_D and FGF23 strongly induce the expression of CYP24A1, while PTH reduces its expression by stimulating its mRNA [72]. Moreover, PTH enhances the production of 1,25(OH)_2_D, which in turn activates an inhibitory loop regulating PTH production. Similarly, FGF23 regulates the production of 1,25(OH)_2_D, an inducer of FGF23 synthesis in the bones [70,73]. These factors work together to maintain vitamin D nutritional homeostasis, explaining why exercise cannot adequately elevate the level of 25(OH)D. Interestingly, serum calcium and PTH levels were significantly increased in the three groups [35]. Changes in PTH and calcium levels may therefore be responsible for the transient changes in 25(OH)D levels when there is no deficiency.

Endurance exercise can increase VDR mRNA levels [38,51,55]. In the target tissue, 1,25(OH)_2_D can bind to VDR and exert physiological functions, which may explain why exercise promotes health. Because 1,25(OH)_2_D utilization in the target tissue increases, so does conversion of 25(OH)D to 1,25(OH)_2_D_3_, resulting in reduced serum 25(OH)D levels [38].

**Figure 1:**
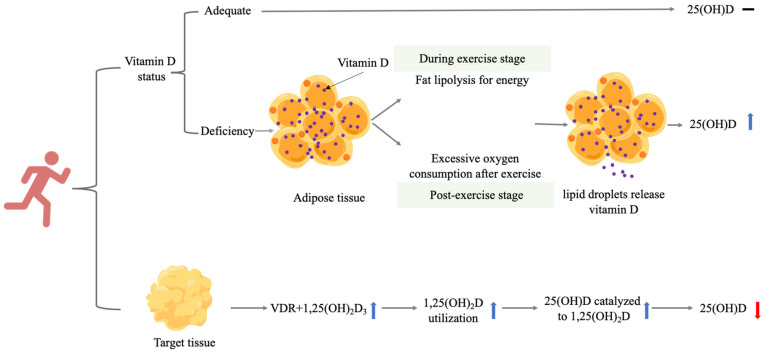
Possible mechanisms of the effect of endurance training on serum 25(OH)D. Vitamin D indicates serum vitamin D metabolites; **−** indicates no significant change; **↑** indicates significant increase; and **↓** indicates significantly reduction. Vitamin D status: 25(OH)D ≥ 20 ng/mL or 50 nmol/L; vitamin D deficiency: 25(OH)D < 20 ng/mL or 50 nmol/L. Abbreviations: VDR, vitamin D receptor.

A summary of how endurance training may exert its effects on 25(OH)D in several ways can be seen in Figure 1.

## 3. Resistance Exercise

### 3.1. The Effect of Acute Resistance Exercise

#### Human and Animal Studies

One human study and one animal study have investigated the effect of acute resistance exercise intervention on 25(OH)D (Table 2). Barker et al. found that serum 25(OH)D concentrations significantly increased immediately after acute resistance exercise in 14 recreationally active adults; however, the levels subsequently decreased after 24, 48, 72, and 168 h [74]. Conversely, Makanae et al. reported no significant change in serum 25(OH)D concentrations in adult male Sprague-Dawley rats in response to acute resistance exercise [37]. In the human study, subjects performed an intense-stretch shortening contraction (10 sets of 10 repetitive jumps), whereas rats were put through isometric exercise (5 sets of 10 contractions). The inconsistency in outcome between the two studies may partially be explained by the differences in resistance exercise intensity and volume.

### 3.2. The Effect of Chronic Resistance Exercise Training

#### 3.2.1. Human Studies

Five human studies have investigated the effects of resistance exercise training on 25(OH)D levels (Table 2). Resistance exercise training significantly increased circulating 25(OH)D levels in vitamin D-deficient post-stroke hemiplegia patients [75] and healthy participants [76]. Conversely, resistance exercise training had no effect on 25(OH)D levels in healthy vitamin D-deficient young men [77] and older adults without vitamin D supplementation [78]. However, Agergaard et al. found that resistance exercise training significantly reduced serum 25(OH)D levels in young and elderly participants without vitamin D supplementation [79]. Factors such as vitamin D supplementation, season, and experimental design should be considered when interpreting these findings. We found that all groups received vitamin D supplementation in Zhang’s study [75]; however, serum 25(OH)D levels were higher at 3 months and 1 year following resistance exercise training combined with vitamin D, compared to only vitamin D supplementation. In Bass’s study, there was a lack of control groups and seasonal information; hence, it is unclear whether the increase in serum 25(OH)D levels is due to resistance training or seasonal factors [76]. Detailed seasonal information was provided in Aschauer’s study (from mid-February to mid-July) [78], Sun’s study (from March to July) [77], and Agergaard’s study (from November to December) [79]. We found a clear seasonal trend in mean serum 25(OH)D concentrations, suggesting that the change in 25(OH)D concentrations induced by resistance training may have been caused by large seasonal fluctuations [77,78].

#### 3.2.2. Animal Studies

Two animal studies have investigated the effect of resistance exercise training on 25(OH)D and 1,25(OH)_2_D_3_ (Table 2). Buskermolen et al. found that 6 weeks of peak power training did not alter serum 25(OH)D levels in rats [52]. Conversely, Xu et al. found that 8 weeks of jumping training significantly reduced serum 1,25(OH)_2_D_3_ levels in male mice [56].

### 3.3. Mechanisms

The predominant adaptation of resistance exercises is in the musculoskeletal system, including increases in muscle mass, muscle strength, and bone density [61]. Muscle mass is increased when resistance exercise triggers muscle signaling events that activate mTOR, leading to increased protein synthesis [62]. Therefore, resistance exercise can be effective in increasing muscle weight and hypertrophy. Mason et al. reported that circulating VDBP can be internalized into skeletal muscle cells to provide high-affinity intracellular binding sites for 25(OH)D [80]. The authors postulate that this intracellular VDBP enables 25(OH)D to diffuse into muscle cells where it is bound and retained until VDBP undergoes proteolysis [80]. The released 25(OH)D then diffuses from the skeletal muscle cells into the circulation and is immediately bound by VDBP in the circulation [80]. Thus, muscle tissue may be an important target tissue and extravascular storage pool for vitamin D. In Sun’s study, fat-free mass and muscle mass were significantly increased [77]. Similarly, in Agergaard’s study, the cross-sectional area of the quadriceps muscle had significant gains in the group who did not receive vitamin D supplements [79]. This result suggests that increased muscle mass from resistance training provides a reservoir of vitamin D, leading to reduced or unchanged serum 25(OH)D levels.

Moreover, 25(OH)D can be released from skeletal muscle [69,81,82,83,84]. This release is regulated by the VDR, PTH, VDBP, and vitamin D nutritional status [69,83,84]. PTH reduces the net uptake of 25(OH)D_3_ in C2 myotubes and mouse muscle fibers and reduces its retention in myotubes [69]. In Barker’s study, PTH levels significantly increased after acute resistance exercise [74]. In Zhang’s study, there was a significant increase in PTH levels at 3 months and 1 year following chronic resistance exercise combined with vitamin D supplements, compared to only receiving vitamin D supplements [75]. The increase in circulating 25(OH)D levels may be due to the effect of PTH on its uptake and retention in skeletal muscle cells [74,75]. However, chronic resistance training alone did not significantly alter the PTH levels [52,77]. This may also be the reason why vitamin D supplementation combined with resistance training, and not resistance exercise training alone, increases 25(OH)D levels.

Resistance training can increase the level of CYP27B1 [37], which can catalyze the conversion of 25(OH)D to 1,25(OH)_2_D_3_. Moreover, resistance training can increase target tissue VDR levels [37], increasing 1,25(OH)_2_D_3_ utilization, which may explain why serum 25(OH)D levels are not altered in response to resistance training [37]. In addition, resistance training increases CYP24A1 levels [56], which can degrade 25(OH)D and 1,25(OH)_2_D; hence, the decrease in 1,25(OH)_2_D may be caused by increased degradation, while its synthesis remains unchanged [56]. These factors may individually or together contribute to reduction/unchanged 25(OH)D levels in the circulation in response to resistance training.

**Figure 2:**
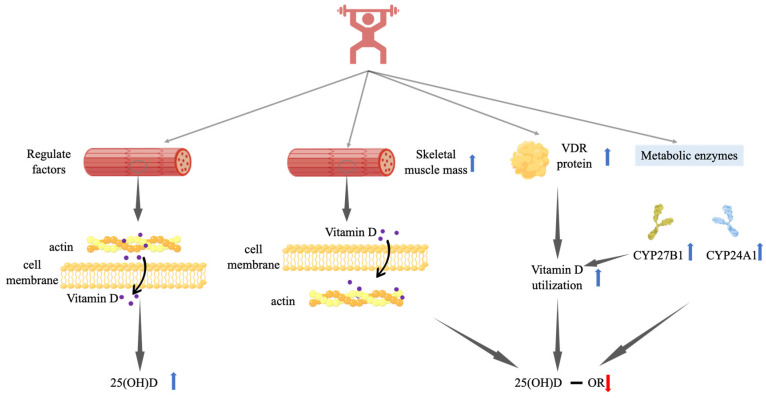
Possible mechanisms of the effect of resistance training on serum 25(OH)D. Vitamin D indicates serum vitamin D metabolites; **−** indicates no significant change; **↑** indicates significant increase; and **↓** indicates significantly reduction. Regulation factors include VDR, PTH, DBP, and vitamin D nutritional status. 25(OH)D-1α hydroxylase, CYP27B1, can convert 25(OH)D to 1,25(OH)_2_D. Vitamin D 24-hydroxylase, CYP24A1, is an important degrading enzyme of vitamin D. Abbreviations: VDR, vitamin D receptor.

How resistance training may exert its effect on 25(OH)D in various ways is briefly illustrated in Figure 2.

## 4. Others

The effect of endurance combined with resistance exercise training intervention on 25(OH)D was investigated in three human studies and one animal study, which did not yield consistent results (Table 3). In the human studies, chronic endurance combined with resistance exercise training intervention significantly increased serum 25(OH)D levels [85,86]. Evans et al. found that 4 months of recruit training significantly reduced serum 25(OH)D levels in healthy men with adequate vitamin D {25(OH)D ≥ 20 ng/mL or 50 nmol/L [57]}, while no significant change was observed in healthy women with adequate vitamin D levels [87]. Conversely, Buskermolen et al. reported that 6 weeks of peak power combined with endurance training did not alter serum 25(OH)D levels in Wistar rats [52].

## 5. Limitations and Perspectives

Aside from the small number of relevant studies, there are many limitations. First, while mass spectrometry, enzyme-linked immunosorbent assays, and other methods can detect 25(OH)D levels, their accuracies vary greatly. Moreover, 25(OH)D_2_ levels are difficult to detect [24]. Second, the vitamin D nutritional status is affected by exposure to season/sunlight. Except for the Sun study and the Li study, which clearly stated that chronic exercise intervention was conducted indoors [77] or outdoors [86], the vast majority of studies did not provide relevant information. Some studies did not provide seasonal information or information on sunlight exposure. Third, some studies did not include a blank control group. These limitations should be addressed in future research. In this paper, there are also some strengths. First, we relatively comprehensively summarize the relevant research in recent years. Second, due to the different effects of different exercise types on health, we focus on the analysis of the effects of endurance training and resistance training exercise. Third, from the perspective of the two major extra-circulating depots and the regulatory factors of vitamin D, this review comprehensively explained the possible mechanism of exercise on vitamin D.

Because the nutritional status of vitamin D is influenced by various factors, we recommend incorporating the following considerations in future studies. First, due to their lipid solubility, vitamin D metabolites are sequestered in adipose tissue, leading to decreased bioavailability in obese subjects [88]. Moreover, Drincic believed that because of volumetric dilution, obese individuals have lower 25(OH)D concentrations [89]. Therefore, body fat is significantly negatively correlated with serum 25(OH)D levels [90] and obese individuals have a higher risk of vitamin D deficiency [91,92]. In addition, lipolysis may be impaired in obese individuals [93], and obesity affects the regulation of vitamin D metabolism enzymes [94], which may explain why 25(OH)D levels in overweight and obese adults were not altered in the Lithgow study [48]. Second, exercise in the fed and fasted states differed in terms of energy metabolism substrates. A study found that exercise performed in the fasted state induces higher fat oxidation than exercise performed in the fed state [95]. Moreover, fasting increases post-exercise circulating FFAs [96]. Therefore, the effects of exercise on serum 25(OH)D or 1,25(OH)_2_D levels may be influenced by whether it is performed under fed or fasted states. Third, vitamin D metabolites are primarily found in circulation, adipose tissue, and skeletal muscle [63]. Thus, adipose and muscle tissues are two major extra circulatory depots for vitamin D metabolites, which are not reflected in serum 25(OH)D levels. Therefore, when studying the effect of exercise on vitamin D, extravascular storage tissues should be included in the analysis.

## 6. Conclusions

**Figure 3:**
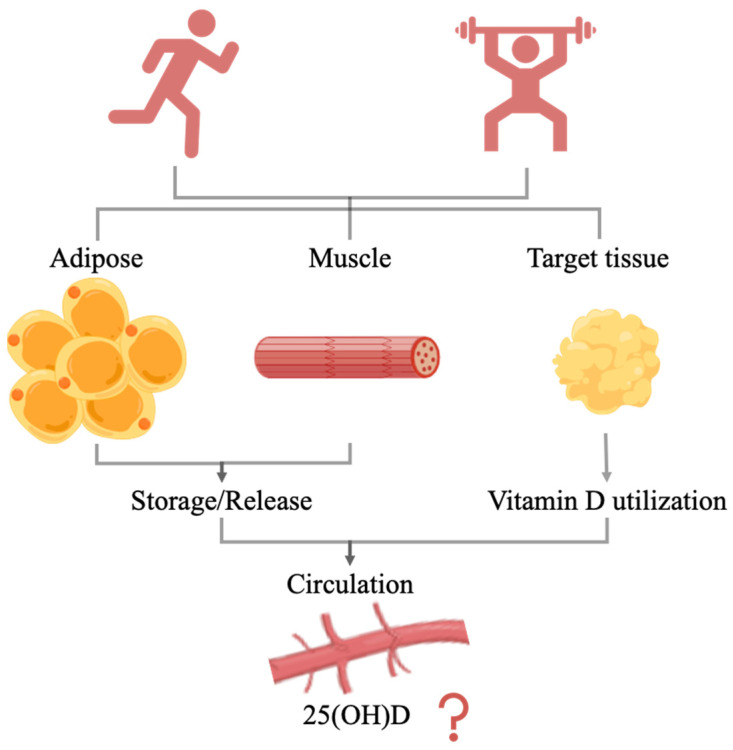
The effect of exercise on 25(OH)D in circulation.

In conclusion, endurance exercise can significantly increase serum 25(OH)D levels in vitamin D-deficient subjects but has no significant effect on vitamin D-sufficient subjects. Moreover, resistance training did not significantly increase 25(OH)D concentrations. Only chronic endurance exercise intervention significantly increased serum 1,25(OH)_2_D levels, and this effect may be sex-dependent. Exercise may influence 25(OH)D levels in circulation by regulating either the release of vitamin D metabolites from storage tissues or the utilization of target tissue (Figure 3). The effects of exercise on 25(OH)D levels may depend on the vitamin D nutritional status, exercise type, exercise intensity, and sex. The organism is a complex entity, and vitamin D is tightly regulated by a variety of factors. Exercise elicits various bodily responses, and the effects of exercise on vitamin D nutritional levels may be the result of a combination of these. Therefore, further research on the effects and mechanisms of exercise on 25(OH)D levels is needed.

## Figures and Tables

**Table 1:** Summary of the effect of acute endurance exercise intervention.

Study	Participants/Animal, *n*	Endurance Exercise Intervention	Sunlight Exposure	Main Findings
Acute endurance exercise intervention-human studies				
Mieszkowski (2020) [32]	Experimental, *n* = 13, 42.00 ± 8.44 years old, Ultra-Marathon Race, 150,000 vitamin D_3_; Control, *n* = 14, 40.00 ± 8.11 years old, Ultra-Marathon Race, placebo solution	Ultra-Marathon Race	18:00 h, 19 July; most of the time, the sky was overcast	25(OH)D_3_: significantly increased immediately and 24 h after the ultra-marathon vs. 24 h before the ultra-marathon in both groups
Dzik (2022) [33]	Male soccer players, *n* = 12 (pre-pubertal, *n* = 5; pubertal, *n* = 7)	VO_2_max test	-	25(OH)D_3_: significantly increased at 15 min and 1 h after exercise vs. before; increased 15 min after the VO_2_max test and dropped one hour after exercise, but not significantly different.
Maimoun (2006) [34]	Male competitive road cyclists, *n* = 7, 20–30 years old	47% Wmax; 64% Wmax	-	25(OH)D: no change 1,25(OH)_2_D: no change
Maimoun (2009) [35]	Elderly moderately active (ModEl, *n* = 18), 71.9 ± 7.3 years old; Elderly active (HAcEl; *n* = 18), 71.7 ± 8.6 years old; Young active (AcYo; *n* = 9), 25.8 ± 2.3 years old	maximal incremental exercise	-	25(OH)D: significantly increased in HAcEl, but not in ModEl and AcYo 1,25(OH)_2_D: no change
Sun (2017) [36]	Healthy young men, *n* = 10, 18–22 years old; Healthy young women, *n* = 10, 19–22 years old	cycling exercise for 30 min at 70% VO_2max_	at the laboratory	25(OH)D: significantly greater at 0 h, 1 h, 3 h and 24 h after exercise vs. before exercise; subgroup analysis: significantly increased at 24 h after exercise in women only 1,25(OH)_2_D: no change
Acute endurance exercise intervention: animal studies				
Makanae (2015) [37]	Adult male Sprague–Dawley rats, 10 weeks age	60 min, 25 m/min	at the laboratory	25(OH)D_3_: no change
Puangthong (2021) [38]	Healthy ponies, *n* = 6 (5 geldings, 1 mare), 6.3 ± 2.2 years age	77–93% of HR_max,_ 16.5 ± 1 min, 5.2 ± 0.3 km	at the laboratory	25(OH)D_2_: significantly reduced at 30 min, 1 week, and 3 weeks after high-intensity exercise
Chronic endurance exercise intervention-human studies				
Farag (2019) [39]	Vitamin D plus PA group: *n* = 21, 40.42 ± 5.89 years old, 2000IU/day, endurance PA	Endurance PA: 12 weeks, daily endurance PA, 30 min/day	Either at morning, 7:30 a.m. or afternoon after 3:00 p.m.	25(OH)D: significantly increased
Mieszkowski (2018) [40]	High-intensity interval training group (HI-NW): LD (*n* = 8, 67.37 ± 6.30 years old, 800 IU/day vitamin D_3_), and HD (*n* = 8, 67.63 ± 7.29 years old, 4000 IU/day vitamin D_3_); Moderate-inteensity continuous training group (MI-NW): LD (*n* = 13, 69.08 ± 4.87 years old, 800 IU/day vitamin D_3_) and HD (*n* = 13, 70.85 ± 4.61 years old, 4000 IU/day vitamin D_3_)	Nordic walking training: 12 weeks, two hours, three times a week. HI-NW: 30 s acceleration going uphill,60 s release going downhill for eight time; 70% HR_max_ for 28 min. MI-NW: 60–70 HR_max_ for 40 min	morning hours	25(OH)D_3_: significantly increased in HI-NW with LD and HD group and MI-NW with HD group; no change in MI-NW with LD group.
Prusik (2018) [41]	Experimental group (EG), *n* = 35, 68.4 ± 5.0 years old	EG: Nordic walking training, 12 weeks, three times a week, 60–70% HR_max_ for 45–55 min; 4000 IU/day vitamin D supplement	1 h after breakfast	25(OH)D_3_: significantly increased after 12 weeks of Nordic walking training with vitamin D supplementation; no change after 6 months without training and vitamin D supplementation
Malandish (2020) [42]	Postmenopausal women Exercise group (EX), *n* = 13, 53.36 ± 3.98 years old; Control group (C), *n* = 13, 53.00 ± 3.26 years old	EX: 12 weeks training, 3 sessions per week, 55–60 min per session, 40 min of walking or jogging aerobic exercise on treadmill C: no intervention	-	25(OH)D: significantly increased after exercise vs. before exercise in EX group and compared to C group; no change in C group
Li (2019) [43]	elderly chronic obstructive pulmonary disease patients with osteoporosis, 65–82 years old Experimental group, *n* = 31; Control group, *n* = 31	Experimental group: 12 weeks, 4 times/week, 5 set/session, 5 min/set, 5 min between sets, 75% CPET, 25 min/session. Control group: 12 weeks, 4 times/week, 5 set/session, 5 min/set, 5 min between sets, 50% CPET, 25 min/session.	-	25(OH)D: significantly increased after exercise in experimental group and control group; significantly increased after exercise in experimental group vs. control group after exercise intervention
Song (2014) [44]	postmenopausal women with type II diabetes and osteoporosis Experimental group: *n* = 278, 52.82 ± 5.12 years old; Control group: *n* = 284, 53.26 ± 5.12 years old	Experimental group: 48 weeks, moderate intensity, 20–30 min/time, two times/day, 0.25 ug/day Calcitriol and 600 mg vitamin D supplementation Control group: 0.25 ug/day Calcitriol and 600 mg vitamin D supplementation	-	25(OH)D: significantly increased 24 weeks and 48 weeks after exercise vs. before exercise in experimental group and higher than control group at same time points
Shi (2013) [45]	Patients with osteoporosis, 50–89 years old, *n* = 82 exercise group (*n* = 40); control group (*n* = 42)	exercise group: Wu xing Bone gymnastics, 90 days, 30–45 min/time, two times/day control group: calcium and Calcitriol supplementation	-	25(OH)D: significantly increased after exercise intervention vs. before exercise intervention in exercise group; no change in control group
Klausen (1993) [46]	Male marathon runners, *n* = 9, 41–50 years old	Endurance training: median running distance was 61 km per week, 4 weeks	the months of December and January	25(OH)D_3_: no change at 2 week and 4 week retraining. 1,25(OH)_2_D_3_: significantly reduced at 4 week retraining vs. before retraining
Pilch (2017) [47]	Women, *n* = 17, 57 ± 4.20 years old	Nordic walking training, 6 weeks, three times a week, 90 min/time, 60–70% HR_max_.	morning hours	25(OH)D: significantly reduced after exercise intervention
Lithgow (2018) [48]	Overweight and obese adults Placebo group: *n* = 10, 34 ± 10 years old; Vitamin D group: *n* = 10, 34 ± 9 years old	Placebo group: HIIT intervention, 6 weeks, 3 sessions/week, 10 repetitions of 1 min intervals interspersed with 1 min active recovery at a power output of 50 W. placebo tablets Vitamin D group: HIIT with 4000 IU/day vitamin D_3_	-	25(OH)D_3_: significantly increased in vitamin D group than placebo group; no change between before and after exercise in placebo group
Hossain (2018) [49]	Intervention group: *n* = 7, 14–18 years old; Control group: *n* = 7, 14–18 years old	Intervention group: brisk walking, 12 weeks, 45 min/time, three times a week Control group: no change routine lifestyle	-	25(OH)D: no change in both groups
Sun (2018) [50]	The 5-week endurance exercise training group (ET group), *n* = 10, 66.5–75.3 years old; Sedentary control group (SC group), *n* = 10, 63.8–73.0 years old	ET group: aerobic exercise, 5 weeks, three times per week, 60% VO_2max_ during week 1, 70% during weeks 2 and 3, and 75% during weeks 4 and 5, 30 min for weeks 1 and 2, and 45 min for weeks 3–5 SC group: no intervention	From October to November	25(OH)D: significantly reduced after exercise in SC group; no change in ET group
Chronic endurance exercise intervention: animal studies				
Aly (2016) [51]	Adult male albino, Group I(a): control sedentary, *n* = 15; Group I(b): control exercised, *n* = 15; Group II(a): diabetic sedentary, *n* = 15; Group II(b): diabetic exercised, *n* = 15	Group I(b) and Group II(b): swimming moderate exercise, 4 weeks, 60 min/time, 5 time per week Group I(a) and Group II(a): no intervention	at the laboratory	25(OH)D: significantly increased in Group II(b) vs. Group II(a); no change between Group I(a) and Group I(b)
Buskermolen (2019) [52]	Female wistar rat, 13 weeks old Control group, *n* = 8; Endurance training group (ET), *n* = 10	ET: treadmill running, 6 weeks, 10 min at a speed of 16 m/min without a slope, increased up to 45 min with a speed of 26 m/min on a 10% slope Control group: no intervention	at the laboratory	25(OH)D: no change between ET and control group
Yeh (1989) [53]	Female Sprague-Dawley rats, 75 ± 5 g Exercise group; Pair-fed exercise group; control group;	Exercise group and Pair-fed exercise group: flat-bed treadmill running, 13 weeks, 60 min/time, 5 times per week, 18–25 m/min Control group: no intervention	at the laboratory	25(OH)D: no change in the three groups 1,25(OH)_2_D_3_: significantly increased in Exercise group and Pair-fed exercise group vs. control
Iwamoto (2004) [54]	Female Wistar rats, 6 weeks old, *n* = 20 7 weeks of exercise (7EX), *n* = 5; 7 weeks of sedentary control (7CON), *n* = 5; 11 weeks of exercise (11EX), *n* = 5; 11 weeks of sedentary control (11CON), *n* = 5	7EX and 11EX: running on flat-bed treadmill, 7 weeks or 11 weeks, 60 min/time, 5 time a week 7CON and11CON: no intervention	at the laboratory	1,25(OH)_2_D_3_: significantly increased in 7EX vs. 7CON; significantly increased in 11EX than 11CON
Wang (2018) [55]	Male F344 rats Sedentary young rats (Young), *n* = 9; Sedentary aged rats (Aged), *n* = 9; Aged rats with aerobic exercise training (Aged + EX), *n* = 9	Aged + EX: running treadmill, 12 weeks, 7 times per week, 1 h/time, 10% slope, 8–20 m/min Young and Aged group: no intervention	at the laboratory	1,25(OH)_2_D_3_: slightly increased, not significant
Xu (2019) [56]	C57BL/6 male mice, 5 weeks old Swimming group (group S), *n* = 7; Downhill running group (group R), *n* = 7; Control (group C), *n* = 7	group S: swimming training, 8 weeks, 6 times per week, 50 min/time, 65–70%VO_2max_ group R: downhill running, 8 weeks, 6 times per week, 50 min/time, −9% slope, 0.8 km/h group C: no intervention	at the laboratory	1,25(OH)_2_D_3_: significantly reduced in group S and group R vs. group C

- Indicates no relevant information. Wmax indicates maximal workload; VO_2max_ indicates maximal oxygen uptake. HR_max_ indicates maximal heart rate. Abbreviations: PA indicates physical activity; HR indicates heart rate; NW indicates Nordic walking training; CPET indicates cardiopulmonary exercise test; and HIIT indicates high-intensity intermittent training.

**Table 2:** Summary of the effect of resistance exercise intervention (human study and animal study).

Study	Participants/Animal, *n*	Resistance Exercise Intervention	Sunlight Exposure	Main Findings
Acute resistance exercise intervention: human study				
Barker (2013) [74]	Recreationally active subjects Intense-stretch shortening contraction leg (SSC); Control leg (CON)	SSC: 10 sets of 10 jumps with a 20-s rest between each set at 75% of body mass on one leg only CON: no intervention	December to March; at the laboratory	25(OH)D: significantly increased immediately after acute resistance exercise; decreased after 24, 48, 72, and 168 h
Acute resistance exercise intervention: animal study				
Makanae (2015) [37]	Male Sprague-Dawley, 10 weeks old	Isometrically exercise, five sets of ten 3 s contractions, with a 7 s interval between contractions and 3 min rest intervals between sets	at the laboratory	25(OH)D_3_: no change
Chronic resistance exercise intervention: human study				
Zhang (2017) [75]	patients with post-stroke hemiplegia, 59.58 ± 4.39 years old Experimental group, *n* = 25; Control group, *n* = 25	Experimental group: weight-bearing exercise training, one year, 40 min/time, two times/day. Routine rehabilitation. Calcium and calciferol supplement Control group: Routine rehabilitation. Calcium and calciferol supplement	-	25(OH)D: significantly increased at 3 months and 1 year of intervention in Experimental group vs. before intervention and vs. control group at same time points.
Bass (2020) [76]	Male and female healthy participants, *n* = 37, 48.4 ± 2.6 years old	20 weeks, three times a week, 70% 1 repetition max, single sets of 12 repetitions with 2-min rests between sets of seated chest press, lat pull down, seated lever row, leg extension, seated leg curl, seated leg press, back extension and abdominal curls	-	25(OH)D: significantly increased after exercise intervention
Sun (2020) [77]	healthy men, *n* = 18, 19–39 years old resistance training group (RT), *n* = 9, 24.2 ± 3.1 years old; non-exercise control group (CON), *n* = 9, 26.7 ± 6.2 years old	RT: progressive resistance training, 12 weeks, 2–3 times per week, resistance workload gradually changed from light to heavy CON: no intervention	From March to July, Between 16:30 h and 20:00 h in a gymnasium	25(OH)D: significantly increased after 12 weeks of exercise intervention vs. baseline in both groups; significantly higher at 6 weeks compared with the values at baseline in the CON group, whereas no notable differences were found in the RT group
Aschauer (2021) [78]	Older adults, *n* = 85, 65–85 years old Control group (CON), Placebo, 400 mg calcium/day; Vitamin D_3_ daily group (VDD), 800 IU vitamin D_3_/day, 400 mg calcium/day; Vitamin D_3_ monthly group (VDM), 50,000 IU vitamin D_3_/month, 400 mg calcium/day	Three groups have conducted Resistance training: 10 weeks, twice a week, 60–90 min/session	From mid-February to mid-July	25(OH)D: no change in CON; significantly increased in both VDD and VDM
Agergaard (2015) [79]	Healthy sedentary young and elderly men Young vitamin D group, *n* = 7, 23.3 ± 2.0 years old; Young placebo group, *n* = 10, 22.4 ± 1.8 years old; elderly vitamin D group, *n* = 7, 67.1 ± 2.9 years old ; elderly placebo group, *n* = 10, 66.6 ± 4.2 years old	Four groups have conducted resistance training exercise: 12 weeks, 3 sessions/week, Progressive loading levels	From November to April	25(OH)D: significantly reduced at 0, 2, 6, and 12 weeks in young placebo group vs. at −4 weeks; significantly reduced at 0, 6, and 12 weeks in young placebo group vs. at −4 weeks; significantly increased at 0, 2, 6, and 12 weeks in young vitamin D group and elderly vitamin D group vs. at −4 weeks
Acute resistance exercise intervention: animal studies				
Buskermolen (2019) [52]	Female wistar rat, 13 weeks old peak power training (PT), *n* = 10; Control group, *n* = 8	PT: peak power training, 10 sprints of 15 s in gallop at a maximal attainable velocity on a progressively increasing slope starting at 10% reaching up to 40% by the end Control group: no intervention	at the laboratory	25(OH)D: no change
Xu (2019) [56]	C57BL/6 male mice, 5 weeks old Jumping group (group J), *n* = 7; Control group (group C), *n* = 7	Group J: jumping training, 8 weeks, 6 times per week, 6–7 sets/min, 50 min/time Group C: no intervention	at the laboratory	1,25(OH)_2_D_3_: significantly reduced in group J vs. group C

- Indicates no relevant information.

**Table 3:** Summary of the effect of endurance combined with resistance exercise intervention (human studies and animal study).

Study	Participants/Animal, *n*	Endurance Exercise Intervention	Sunlight Exposure	Main Findings
Endurance combined with resistance exercise intervention: human studies				
Gustafsson (2019) [85]	healthy, pregnant Norwegian women Intervention group: *n* = 429, 30.5 ± 4.4 years old; Control group: *n* = 426, 30.4 ± 4.3 years old	Intervention group: aerobic and strength training, 12 weeks, 3 times per week, 60 min/time	-	25(OH)D: no significant effect of the exercise program on levels of total, free, or bioavailable 25(OH)D in only baseline level adjust model; additionally adjusted for study site and sampling month, revealed a significant between-group difference in levels of total, free, and bioavailable 25(OH)D.
Li (2019) [86]	Patients with postmenopausal osteoporosis Training group: *n* = 26, 55.46 ± 4.12 years old; Control group: *n* = 26, 56.25 ± 3.75 years old	Training group: 12 weeks, (a) endurance exercise training, brisk walk outdoors, 4 times per week, 30 min/ time, 50%VO_2max_; (b) progressive resistance training. calcium and Calcitriol supplementation Control group: calcium and Calcitriol supplementation	brisk walk outdoors	25(OH)D: significantly increased after intervention in both groups; significantly increased in Training group vs. control group
Evans [87]	Healthy men, *n* = 41, 19.3 ± 1.2 years old; Healthy women, *n* = 153, 19.0 ± 1,0 years old	Marching under load, running and jumping, battle drills, and walking and standing for prolonged periods of time	-	25(OH)D: significantly reduced at 4 months in male participants; no change in female participants
Endurance combined with resistance exercise intervention-animal study				
Buskermolen [52]	Female wistar rat, 13 weeks old peak power training and endurance training group, *n* = 10; Control group, *n* = 8	Peak power training: 10 sprints of 15 s in gallop at a maximal attainable velocity on a progressively increasing slope starting at 10% reaching up to 40% by the end endurance training: treadmill running, 6 weeks, 10 min at a speed of 16 m/min without a slope, increased up to 45 min with a speed of 26 m/min on a 10% slope Control group: no intervention	-	25(OH)D: no change

- Indicates no relevant information.

## Data Availability

No new data were created or analyzed in this study. Data sharing is not applicable to this article.
